# The Size-Weight Illusion is not anti-Bayesian after all: a unifying Bayesian account

**DOI:** 10.7717/peerj.2124

**Published:** 2016-06-16

**Authors:** Megan A.K. Peters, Wei Ji Ma, Ladan Shams

**Affiliations:** 1 Department of Psychology, University of California, Los Angeles, CA, United States; 2 Center for Neural Science, New York University, New York, NY, United States; 3 Department of Psychology, New York University, New York, NY, United States; 4 Department of Bioengineering, University of California, Los Angeles, CA, United States

**Keywords:** Size-Weight Illusion, Hierarchical causal inference, Bayesian inference, Heaviness perception

## Abstract

When we lift two differently-sized but equally-weighted objects, we expect the larger to be heavier, but the smaller *feels* heavier. However, traditional Bayesian approaches with “larger is heavier” priors predict the smaller object should feel *lighter*; this Size-Weight Illusion (SWI) has thus been labeled “anti-Bayesian” and has stymied psychologists for generations. We propose that previous Bayesian approaches neglect the brain’s inference process about density. In our Bayesian model, objects’ perceived heaviness relationship is based on both their size and inferred density relationship: observers evaluate competing, categorical hypotheses about objects’ relative densities, the inference about which is then used to produce the final estimate of weight. The model can qualitatively and quantitatively reproduce the SWI and explain other researchers’ findings, and also makes a novel prediction, which we confirmed. This same computational mechanism accounts for other multisensory phenomena and illusions; that the SWI follows the same process suggests that competitive-prior Bayesian inference can explain human perception across many domains.

## Introduction

When we lift two objects of the same mass but differing sizes, we expect the larger to be heavier, but instead the smaller feels heavier. This Size-Weight Illusion (SWI) has been studied from perceptual, computational, and sensorimotor perspectives, but has yet to be satisfactorily explained. One possibility is that observers expect larger objects to be heavier than smaller objects, and so “overlift,” i.e., generate greater force in preparing to and lifting larger objects. Although this would indeed lead the larger object to feel lighter than the smaller (Gordon et al., 1991), in fact motor forces scale quickly and appropriately to the true weight of items while the SWI persists (Buckingham & Goodale, 2010; Flanagan & Beltzner, 2000; Flanagan et al., 2001; Grandy & Westwood, 2006). The SWI also persists when grip size, rotational inertia, and lifting style are controlled (Ellis & Lederman, 1998; Kawai, Henigman & MacKenzie, 2007). Therefore, this illusion cannot depend entirely on motor-system explanations, instead representing a perceptual phenomenon.

Early models that simply described the SWI (e.g., Anderson, 1970; Cross & Rotkin, 1975) do not satisfactorily answer the question of *why* it occurs. Thus, in more recent years, many have reexamined the SWI utilizing Bayesian decision theory: the expectation that smaller objects are lighter than larger ones is formalized as the *prior*, while the sensory evidence that the two objects actually weigh the same is the *likelihood*. The two objects’ heaviness relationship is then represented by the *posterior*, arrived at via Bayes’ rule:

(1) \begin{eqnarray*}p \left( w{|}s \right) = \frac{p \left( s{|}w \right) p \left( w \right) }{p \left( s \right) } .\end{eqnarray*}

Thus, the probability of two objects’ relative weights *w* given their sizes *s*, *p*(*w*|*s*), is a function of the sensory evidence *p*(*s*|*w*) and the prior probability of their weight relationship *p*(*w*) , with *p*(*s*) a normalization constant. This framework has successfully explained numerous perceptual phenomena (e.g., visual motion perception (Weiss, Simoncelli & Adelson, 2002), audiovisual localization (Körding et al., 2007; Wozny, Beierholm & Shams, 2010), and visuohaptic percepts of stiffness versus brightness (Ernst, 2007)). Unfortunately, Bayesian decision theory in this form fails badly at predicting the SWI, instead predicting precisely the opposite: the prior expectation that larger objects are heavier would shift the perception of the larger object to feeling *heavier* than the smaller object (see Supplemental Information, available online, for more detail). For this reason, the illusion has been called “anti-Bayesian” (Brayanov & Smith, 2010). However, such simple formulations neglect the relevance of a factor long-described as critical to the sense of heaviness: density.

Density is not immediately observable, defined only as the relationship between two other properties: volume and mass. It is thus hidden from immediate access for human observers, but has been repeatedly shown to be crucial in heaviness estimation (Buckingham & Goodale, 2013; Cross & Rotkin, 1975; Ross & Di Lollo, 1970). It has also been shown that visual estimates of material affect predictions of an object’s weight (Ellis & Lederman, 1998), and that visual size estimation alone can play a role in density estimation *prior* to lifting an object, such that smaller objects are judged to be denser than larger objects even when visual material is held constant (Peters, Balzer & Shams, 2015). Other investigations have reported that well-learned material-density priors interact with sensorimotor memory of previous lifts in producing heaviness percepts (Baugh et al., 2012), and that a single representation of typical object density based on visual size alone may underlie the SWI (Buckingham et al., 2016; Buckingham & Goodale, 2013).

In contrast, *how* density should computationally affect heaviness percepts has not yet been settled, but causal inference (competitive prior) models may provide some answers. In these models, the perceptual system evaluates the relative probabilities of several competing causal scenarios in addition to evaluating incoming sensory evidence (Yuille & Bülthoff, 1996). These competing scenarios typically reference *secondary* or *hidden* variables (the *cause* of sensory experiences), which the observer does not necessarily estimate explicitly but which nevertheless influence the variables s/he is estimating. For example, in order to interpret the shape of an object, secondary variables must also be evaluated: is the object’s surface material Lambertian (matte) or specular (shiny)? Only once determination about the object’s material has been made do other visual cues to object geometry become interpretable (Yuille & Bülthoff, 1996).

Yuille & Bülthoff (1996) proposed a competitive-hypothesis framework to solve this problem, carving the continuous space of surface reflectance into two categories, Lambertian and specular. A mathematically equivalent framework explains the perception of slant, in which the brain estimates the angle of a slanted plane (Knill, 2003; Knill, 2007) in part by partitioning the continuous space of the hidden variable of possibly isometric, rounded shapes into two qualitative categories: oval and circle. Another version defines as its categorical secondary variable the *cause* of sensory information as a single versus multiple sources: congruent multisensory signals are integrated, while incongruent ones are segregated (Körding et al., 2007), explaining the ventriloquist illusion (Wozny, Beierholm & Shams, 2010), sound-induced flash illusion (Wozny, Beierholm & Shams, 2008), and rubber-hand illusion (Samad, Chung & Shams, 2015). Similarly, in the SWI the estimation of an observed variable (weight) depends on the inference about a secondary unobserved variable (density), and therefore Bayesian causal inference about competing, categorical hypotheses on density may explain perception of weight.

## Materials & Methods

### General behavioral methods

#### General stimuli

Stimuli for all experiments consisted of four sizes of tagboard cubes (three of each size) covered in thin balsa wood, with wooden handles affixed to the top. The cubes were of 5.08, 7.62, 10.16, and 15.24 cm on a side, thus having volumes of 131.10, 442.45, 1048.77, and 3539.61 cm^3^ (cubes A, B, C, and D, respectively). Three sets of differently-weighted cubes (weighted with combinations of steel pellets and cotton) were used, with each set comprised of one cube of each size. Sets thus differed only in weight, so each cube in the Light (L) set weighed 150 grams, those in the Medium (M) set weighed 350 grams each, and those in the Heavy (H) set weighed 550 grams each. Weight was measured with 0.1 gram precision (LB-1000 Scale, American Weigh Scales), which is well below just-noticeable differences (JNDs) for weight perception (between 1.03 and 6.34 g (Kawai, Henigman & MacKenzie, 2007)). Cubes were fitted with handles such that grip size was identical for all cubes in all sets regardless of size and weight.

#### General procedures

All experimental procedures were conducted in accordance with the Declaration of Helsinki and approved by the UCLA Institutional Review Board (UCLA IRB Approval #11-000527).

On each trial, cubes were presented two at a time, placed side by side in front of the participant. The cube to the participant’s left was given a reference weight of 10 units, and the subject was instructed to verbally report his or her perception or expectation regarding the cube on the right, forming a ratio referencing the left cube’s weight. Subjects could experience *Lifting* sessions (Experiments 1 and 2), in which they lifted the cubes sequentially and judge the Perceived Weight (PW) of the second cube, and/or *Expectation* sessions (Experiment 2), in which they guessed the weight of the second cube (Expected Weight, EW) without touching either cube.

In both Expectation and Lifting sessions, cubes were presented in a full factorial design, including all six combinations of the four sizes. Thus, the possible pairings were: A:B, A:C, A:D, B:C, B:D, C:D, (small/left-big/right, S-B); B:A, C:A, D:A, C:B, D:B, D:C (big/left-small/right, B-S). Expectation sessions also included trials in which the two cubes were identically sized as a control: pairs A:A, B:B, C:C, D:D (identically-sized, I-S). In Lifting sessions, following 10 practice trials subjects completed 144 test trials (4 trials of each S-B and B-S pairing). In Expectation sessions, subjects completed 10 practice trials, followed by 128 test trials (8 trials of each S-B and B-S pairing, and 8 trials of each I-S control condition). Box pairs in Lifting sessions were always of the same weight (i.e., it was never the case that the boxes possessed different weights). No feedback was given in either Lifting or Expectation sessions. While the experimenter was placing or removing the cubes, subjects closed their eyes to avoid any cuing effects regarding the possible weight of the cubes. Cubes not in use on a given trial were hidden behind a black curtain. The experimenter also remained hidden from view.

### Experiment 1 methods

#### Participants

Thirty-five healthy participants (mean age: 19.97, range: 18–26, 16 men, 32 right-handed) gave written informed consent to participate in the study. Five subjects (1 man) were excluded due to technical difficulties (stimuli breaking) or noncompliance with experimental procedures or instructions. As a result, 30 subjects participated in this study.

#### Procedure

Behavioral procedures followed the general procedures for Lifting sessions.

#### Statistical analysis

After log transform, the mean response for each condition of interest for each subject was calculated, collapsing across S-B and B-S orderings. We did not collapse across weight class (L, M, H).

### Experiment 2 methods

#### Participants

Thirty-five subjects (mean age: 20.07 years, range: 18–27 years, 14 men, 31 right-handed) gave written informed consent to participate in this study. Five subjects (2 men) were excluded due to technical difficulties (stimuli breaking) or noncompliance with experimental procedures or instructions. As a result, 30 participants participated in this study.

#### Procedure

On Day 1 subjects were instructed that when the two objects were different in size, the smaller one was denser than the larger one; when they were the same size, subjects were instructed to assume they had the same density. No other information was given about the actual density of the items to participants, or about how much denser a smaller object might be than a larger object. Day 1 behavioral procedures followed the general procedures for Expectation sessions: subjects were instructed to provide their Expected Weight (EW) reports without touching or moving the cubes in any way. Day 2 consisted of a Lifting session, in which participants lifted the cubes and provided responses regarding their Perceived Weight (PW).

**Figure 1:**
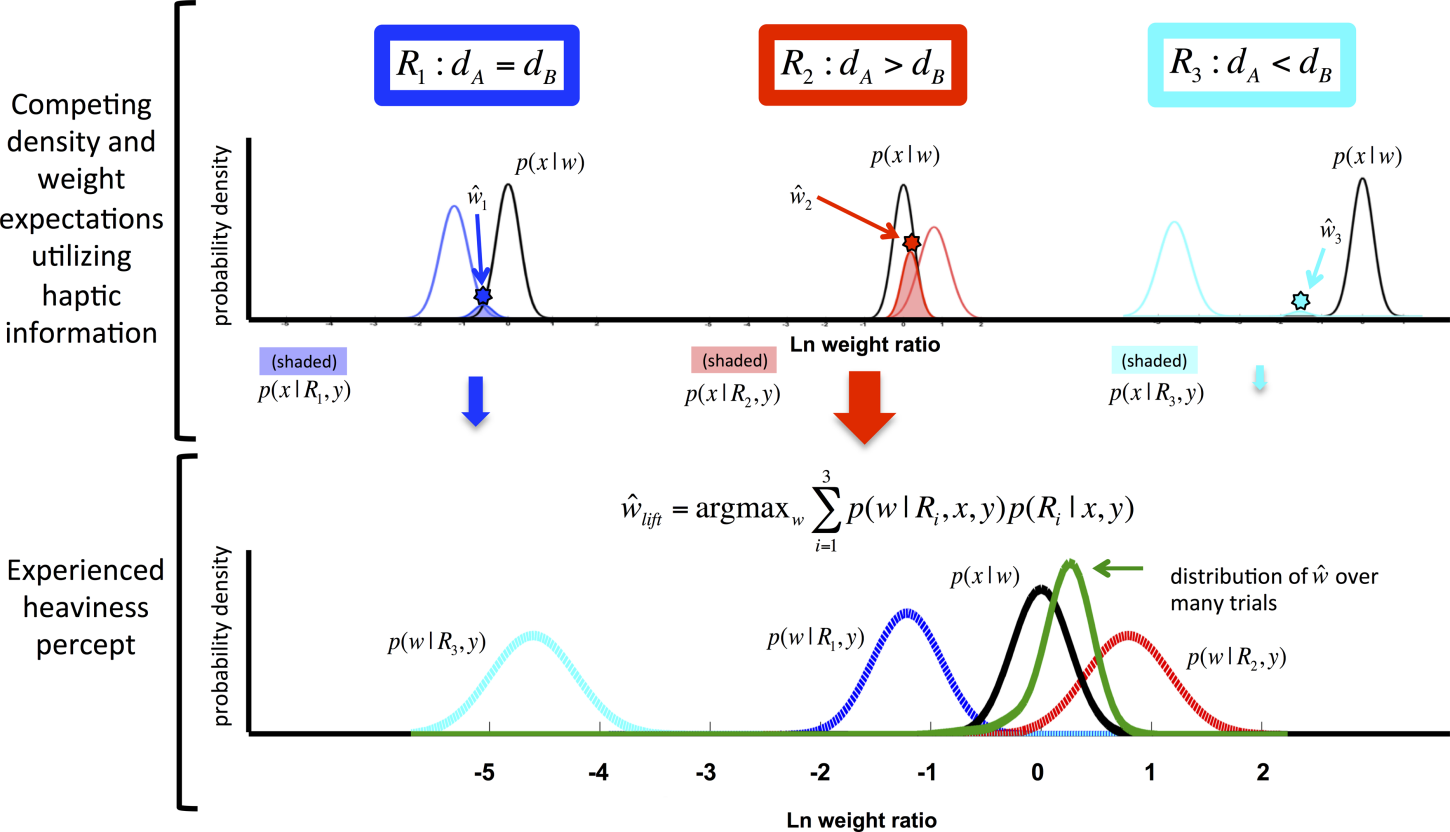
Depiction of the competitive prior framework. The “agreement” between the sensory evidence *p*(*x*|*w*) and competing expectations of *w*—depicted by the shaded regions, or *p*(*x*|*R*_*i*_, *y*)—is greater for *R*_2_ than for the other *R*s. When *p*(*x*|*R*_2_, *y*) is multiplied by the a priori probability of *R*_2_ to produce *p*(*R*_2_|*x*, *y*), it is clear that the expected weight relationship between the two objects under *R*_2_ will exert the most influence on the ultimate percept, $\hat {w}$. In log space, values above 0 represent a felt or expected weight ratio in the SWI range (smaller is heavier). For an extended version of the graphical depiction, see the Supplemental Information.

#### Statistical analysis

Due to the nature of the dependent measure as a ratio (and in keeping with studies on relative mass in intuitive physics (Sanborn, Mansinghka & Griffiths, 2013)), the log transform of each data-point was computed, as was the mean log ratio for each subject for each cube pair. Following log transform, the mean EW response for each condition of interest for each subject was calculated, collapsing across S-B and B-S orderings, and excluding I-S control pairings. We then additionally collapsed across weight classes to determine the mean SWI magnitude (ln(PW)) for each pair, and correlated the ln(EW) and ln(PW) responses for each pair produced by each subject.

### Computational model

To apply the causal inference concept to the SWI, we assume that the brain evaluates the relative probabilities of three “density relationships” (*R*) given incoming haptic information of two objects’ weights, and then evaluates that incoming sensory information in light of the inferred density probabilities (Fig. 1). This density relationship between two objects *A* and *B* with volume relationship *V*_*A*_ < *V*_*B*_ can belong to one of three categories: (*R*_1_) *d*_*A*_ = *d*_*B*_ (e.g., the same material); or (*R*_2_) *d*_*A*_ > *d*_*B*_; or (*R*_3_) *d*_*A*_ < *d*_*B*_ with *d* denoting the density of each object. Note that *R*_2_ and *R*_3_ are qualitative relationships only (Knill, 2003; Knill, 2007; Yuille & Bülthoff, 1996), and do not specify how much the densities will differ.

The SWI model begins with a joint probability over volume and density ratios which varies by density relationship *R*, *p*(*v*, *d*|*R*). When paired with visual estimation of volume, these priors give rise to expectations of weight, which are compared to and combined with the haptic sensation of weight (Fig. 1). In our experiments, participants report a value representing the felt heaviness of two objects in the form of a ratio (see ‘Materials & Methods’: ‘General behavioral methods’). So, because the state variable of interest in our model, *w*, is a ratio, measurement noise can be assumed to be log-normal. (See Table S1 for graphical depiction of the generative model.)

#### Priors

For each density relationship *R*, we define a prior over the joint probability of volume ratios $v=\ln \frac{{V}_{A}}{{V}_{B}} $ and density ratios $d=\ln \frac{{d}_{A}}{{d}_{B}} $. As in prior research (Knill, 2003; Yuille & Bülthoff, 1996), we assume that the nervous system represents the continuous space of density relationship categorically, for computational efficiency. We therefore assume the space of joint probabilities of volume and density can be represented by three bivariate Gaussian distribution with *μ* = [0, 0] and each having ${\mrm{\Sigma }}_{i}= \left[ {\scriptsize \begin{array}{@{}ll@{}} \displaystyle {\sigma }_{v}^{2}&\displaystyle {\rho }_{i}{\sigma }_{v}{\sigma }_{d,i}\\ \displaystyle {\rho }_{i}{\sigma }_{v}{\sigma }_{d,i}&\displaystyle {\sigma }_{d,i}^{2} \end{array}} \right] $, with *ρ*_*R*_2__ < 0 and *ρ*_*R*_3__ > 0 such that

(2) \begin{eqnarray*}p \left( v,d{|}{R}_{i} \right) =N \left( v,d; \left[ v=0,d=0 \right] ,{\mrm{\Sigma }}_{i} \right) \hspace*{10.00002pt}\text{with}~{\sigma }_{d,2}={\sigma }_{d,3}~\text{and}~{\sigma }_{d,1}=0.\end{eqnarray*}

We then define a joint prior as the sum of each of these prior distributions weighted by their a priori probabilities (for each volume relationship), i.e.,

\begin{eqnarray*}p \left( v,d \right) =p \left( v,d{|}{R}_{1},y \right) p \left( {R}_{1},y \right) +p \left( v,d{|}{R}_{2},y \right) p \left( {R}_{2},y \right) +p \left( v,d{|}{R}_{3},y \right) p \left( {R}_{3},y \right) \end{eqnarray*}

(3) \begin{eqnarray*}\hspace*{10.00002pt}\text{with}~p \left( {R}_{1},y \right) +p \left( {R}_{2},y \right) +p \left( {R}_{3},y \right) =1.\end{eqnarray*}

This joint probability of a density relationship and volume relationship *p*(*R*, *y*) is defined in line with previously reported data (Peters, Balzer & Shams, 2015).

#### Incoming sensory evidence

Upon seeing the two objects placed side by side, the brain estimates their volume ratio. It is known that volume is systematically underestimated by an exponent of ∼0.704, i.e., that ${\hat {V}}_{average}\approx {V}^{ \left( .704 \right) }$ (Frayman & Dawson, 1981), therefore we set the mean of the visual volume measurement to be ${v}^{\ast }=\ln { \left( \frac{{V}_{A}}{{V}_{B}} \right) }^{ \left( .704 \right) }$. We then define visual variance ${\sigma }_{y}^{2}$, giving $p \left( y{|}v \right) =N \left( y;{v}^{\ast },{\sigma }_{y}^{2} \right) $. Likewise, we define the haptic estimate of the weight ratio *x* to be distributed normally, with mean $w=\ln \frac{{w}_{A}}{{w}_{B}} =0$ as the estimate of weight ratio should be unbiased, and variance ${\sigma }_{x}^{2}$, such that $p \left( x{|}w \right) =N \left( x;w=0,{\sigma }_{x}^{2} \right) $ . Note that $w=\ln \frac{{w}_{A}}{{w}_{B}} =0$ denotes cases where the objects physically weigh the same; for other weight ratios, the haptic estimate mean changes accordingly.

#### Competing hypotheses

The posterior probability of the log weight ratio under each scenario *R*_*i*_ is computed using Bayes’ Rule as follows:

(4) \begin{eqnarray*}p \left( w{|}R,x,y \right) \propto p \left( w,x,y{|}R \right) =p \left( x{|}w \right) \iint p \left( y{|}v \right) p \left( w{|}v,d \right) p \left( v,d{|}R \right) dvdd.\end{eqnarray*}

Because the weight of an object is deterministically defined by the combination of its volume and density, we take the probability of a weight relationship between two objects given their density and volume relationships, *p*(*w*|*v*, *d*), to be a delta function, $\delta \left( w- \left( d+v \right) \right) $, which is 0 at all impossible combinations of volume and density for a given weight relationship. This posterior mean of the log weight ratio under density hypothesis *R*_*i*_ will be:

(5) \begin{eqnarray*}{\hat {w}}_{i}=\int wp \left( w{|}x,y,{R}_{i} \right) dw.\end{eqnarray*}

When no haptic information is available, the estimate of the weight ratios is computed as the maximum a posteriori (MAP) estimate:

(6) \begin{eqnarray*}{\hat {w}}_{noLift}={\arg \max }_{w}\sum _{i=1}^{3}p \left( w{|}{R}_{i},y \right) p \left( {R}_{i},y \right) .\end{eqnarray*}

In the case where haptic information *is* available, this information is also used in the process of arbitration among the competing hypotheses. The posterior probability of each causal scenario *R*_*i*_ is therefore calculated according to Bayes’ Rule as follows:

(7) \begin{eqnarray*}p \left( R{|}x,y \right) = \frac{p \left( x{|}R,y \right) p \left( R,y \right) }{p \left( x \right) } \end{eqnarray*}

with the likelihood (the probability of a haptic estimate given a density relationship and volume relationship) *p*(*x*|*R*, *y*) obtained via:

(8) \begin{eqnarray*}p \left( x{|}R,y \right) =\int p \left( x{|}w \right) p \left( w{|}R,y \right) dw\end{eqnarray*}

with *p*(*w*|*R*, *y*) equivalent to *p*(*w*|*R*, *x*, *y*) with no haptic information present, as described above. The system’s optimal estimate of the felt weight ratio, $\hat {w}$, is found through the MAP estimate as before:

(9) \begin{eqnarray*}{\hat {w}}_{lift}={\arg \max }_{w}\sum _{i=1}^{3}p \left( w{|}{R}_{i},x,y \right) p \left( {R}_{i}{|}x,y \right) .\end{eqnarray*}

We estimate most parameters of the model according to findings in the literature (see below). We are then left with the following parameters: the visual variance, ${\sigma }_{y}^{2}$; the haptic variance, ${\sigma }_{x}^{2}$; the elements of the covariance matrix governing the a priori relationship between volume and density for each of the density relationships *R*, ${\mrm{\Sigma }}_{i}= \left[ {\scriptsize \begin{array}{@{}ll@{}} \displaystyle {\sigma }_{v}^{2}&\displaystyle {\rho }_{i}{\sigma }_{v}{\sigma }_{d,i}\\ \displaystyle {\rho }_{i}{\sigma }_{v}{\sigma }_{d,i}&\displaystyle {\sigma }_{d,i}^{2} \end{array}} \right] $; and the a priori probabilities for each density-volume relationship *R*, with $p \left( {R}_{1},y \right) +p \left( {R}_{2},y \right) +p \left( {R}_{3},y \right) =1$.

#### Setting model parameters

We make the following assumptions about model parameters, although the presence of the SWI is robust to most reasonable parameter values. For the simulations, we set ${\sigma }_{y}^{2}=0.10$, ${\sigma }_{x}^{2}=0.50$, ${\sigma }_{v}^{2}=0.85$, ${\sigma }_{d}^{2}=1.2$, *ρ*_*R*_2__ = − 0.95, *ρ*_*R*_3__ = 0.95, *p*(*R*_1_, *y*) = 0.8, *p*(*R*_2_, *y*) = 0.15, and *p*(*R*_3_, *y*) = 0.05. These a priori probabilities *p*(*R*, *y*) for the three *R* relationships are qualitatively consistent with the everyday object data collected by Peters, Balzer & Shams (2015), with the change that in the real world objects are rarely the same density if they have different sizes. However, in SWI studies the material of the objects is visually similar, which artificially inflates the probability that they will have the same density. (Note that this would not be the case under the *material-weight illision*, in which objects’ visual material is manipulated; see also the ‘Discussion’ for more). The model is extremely robust to perturbations of these parameter estimates, with the illusion occurring even when $0.1\lt {\sigma }_{x}^{2}\lt 0.7$, $0.05\lt {\sigma }_{y}^{2}\lt 0.7$ (with the exception that at ${\sigma }_{y}^{2}=0.05$ the illusion does not occur for pair BC, or the two objects that are closest in volume), $0.9\lt {\sigma }_{d}^{2}\lt 2$, $0.6\lt {\sigma }_{v}^{2}\lt 1.1$, 0.75 < *ρ* < 1 (with the exception that at *ρ* = 0.75 the illusion does not occur for pair BC), and many different combinations of [*w*_1_, *w*_2_, *w*_3_]. These values should not be taken as the limits of the possible parameters, but as illustration of the range over which the model is extremely robust to reasonable perturbations of parameter values.

## Results

According to the proposed model (see ‘Materials & Methods’: ‘Computational model’), the relative probabilities of three competing “density relationships” *R* are first evaluated in light of incoming visual and haptic information, and their relative probabilities are then used to arrive at the percept of heaviness. For two objects *A* and *B*, where *A* is smaller than *B* (*V*_*A*_ < *V*_*B*_), these density relationships are: the objects have equal density (*R*_1_: *d*_*A*_ = *d*_*B*_); the smaller object is denser (*R*_2_: *d*_*A*_ > *d*_*B*_); or the larger object is denser (*R*_3_: *d*_*A*_ < *d*_*B*_); with *d* denoting each object’s density. The weight relationship between the two objects in the form of a ratio, $ \frac{{w}_{A}}{{w}_{B}} $, can then be estimated based on the inferred density relationship. $ \frac{{w}_{A}}{{w}_{B}} \gt 1$ indicates the presence of the SWI (the smaller object feels heavier). In the behavioral experiments, we measured the magnitude of the SWI in two groups: one group to provide a pure estimate of SWI magnitude, and another to test the model’s predictions. Data are available online.

### Experiment 1: measurement of Size-Weight Illusion magnitude

We quantitatively measured the magnitude of the SWI for various size/weight conditions. Participants lifted pairs of objects and reported their felt heaviness ratio. Data from this experiment may onto the distribution of $\hat {w}$ in the model.

**Figure 2:**
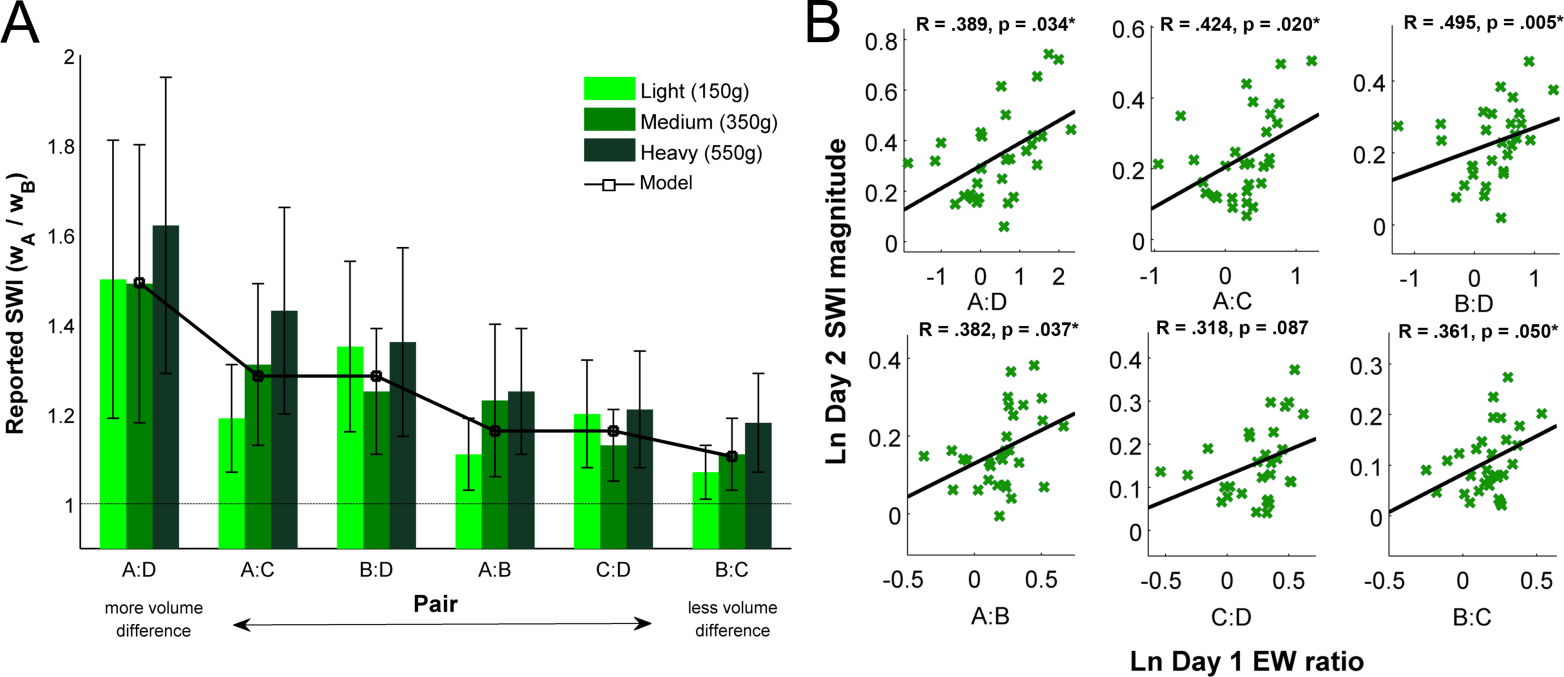
(A) Means of felt heaviness responses in Experiment 1 (green), compared to model predicted SWI magnitudes (black). Although analyses were done on log-transformed data, these results are presented in ratio form for ease of interpretation. Values above 1 indicate illusion. Error bars denote the standard error of the mean. (B) Correlation between Day 1 predicted density asymmetry and Day 2 felt heaviness ratios, as predicted by the model.

A 3 (weight class: L, M, H) × 6 (pair: A:B, A:C, A:D, B:C, B:D, C:D) repeated measures ANOVA revealed the expected main effect of box pair (*F*(5, 145) = 110.282, *p* < .001) indicating the larger the size difference between the two cubes, the larger the illusion, and an additional main effect of weight class (*F*(2, 58) = 16.974, *p* < .001) indicating SWI magnitude is significantly larger the heavier the objects’ weight. Further, an interaction was revealed between weight class and pair (*F*(10, 290) = 8.777, *p* < .001), showing that the amount by which size difference (i.e., pair) influences SWI magnitude increases as weight increases (Fig. 2A). These results mirror the oft-reported finding that the SWI grows with more discrepant size differences between objects (Buckingham & Goodale, 2013; Cross & Rotkin, 1975; Flanagan & Bandomir, 2000; Flanagan & Beltzner, 2000; Flanagan, Bittner & Johansson, 2008). Although there is a difference in mean illusion strength as a function of weight class, there is no consistent pattern in the variability of subjects’ responses. The magnitude of the illusion is also quite consistent across individuals in the current study, with the average between-subjects variability for the mean response to each size-weight condition (*σ* = 0.1214) smaller than the average within-subjects variability across multiple trials of the same condition (*σ* = 0.2103).

### Model results

The model’s predicted reports under Expectation and Lifting conditions were made with the same parameter settings under both conditions (${\sigma }_{y}^{2}=0.10$, ${\sigma }_{x}^{2}=0.50$, ${\sigma }_{v}^{2}=0.85$, ${\sigma }_{d}^{2}=1.2$, *ρ*_*R*_2__ = − 0.95, *ρ*_*R*_3__ = 0.95, *p*(*R*_1_, *y*) = 0.8, *p*(*R*_2_, *y*) = 0.15, and *p*(*R*_3_, *y*) = 0.05), with the exception that the Expectation condition was assumed to have infinite haptic variance (i.e., no haptic information is present, or ${\sigma }_{x}^{2}=\infty $). Crucially, without any haptic information, the model reasonably and expectedly predicts that the estimated weight relationships ought to closely match visually estimated volume relationships: from smallest volume ratio to largest, the predicted weight ratios without lifting are [0.01, 0.23, 0.23, 0.43, 0.43, 0.55].

Model predictions for Lifting sessions are shown in Fig. 2A, superimposed on the data collected in Experiment 1. As can be seen the model provides remarkably good explanatory power for the measured SWI magnitudes, and the expected SWI magnitude is robust to reasonable perturbations in parameter value selection. The model demonstrates the well-known finding that illusion magnitude grows with larger size differences between the two items, which is derived from the oft-reported finding that perceived heaviness decreases as a power function of volume with weight held constant (Cross & Rotkin, 1975; Flanagan & Beltzner, 2000; Flanagan et al., 2001; Grandy & Westwood, 2006; Ross & Di Lollo, 1970; Stevens & Rubin, 1970). Thus, these results demonstrate that a Bayesian model can capture the SWI.

#### Model prediction 1: effect of exposure to unusual density relationships on the illusion

Due to its competitive prior nature, the model predicts that if an alteration is made to the distribution of expected weight relationships under *R*_2_ (smaller is denser) such that this probability distribution becomes increasingly incongruous with incoming sensory information, the other two competing density relationships will become relatively more probable a posteriori. This is because under *R*_2_ (“smaller is denser”) with default (everyday) priors, the model predicts that a smaller, denser object will be denser by a factor of two or three. However, if via training this prior is altered such that a smaller, denser object is expected to be denser by a factor of 10 or even 100, the expected weight relationship under *R*_2_ will bear no resemblance to the (noisy) haptic evidence centered at $ \frac{{w}_{A}}{{w}_{B}} =0$. Because the remaining scenarios predict that the large item will be heavier (*R*_1_; “equal density”) or *much* heavier (*R*_3_; “larger is denser”) than the smaller, their growing relative agreement with the incoming sensory information predicts attenuation and ultimately reversal of the SWI. This prediction matches the behavioral finding reported by Flanagan and colleagues (2008), in which training with “inverted” objects ultimately reverses the SWI. Although the magnitude of the reversal predicted by the model may change with differing assumptions (e.g., about the effect of training on mean and variance of expected weight ratios under *R*_2_), logically the reversal occurs eventually for any pair of objects. And indeed, the reversal may never reach the strength of the original SWI itself, as *R*_2_ will continue to exert influence even if it becomes less probable; this qualitative trend matches the behavioral findings reported by Flanagan and colleagues (2008). More studies should be done to measure the exact influence on the *R*_2_ prior with such inverted object training and to make quantitative predictions.

#### Model prediction 2: effect of individual differences in density expectations

According to our model, the degree of the SWI depends partly on the assumed density of the two objects ($ \frac{{d}_{A}}{{d}_{B}} $). The model predicts that as the assumed density asymmetry increases illusion magnitude should also increase in most cases: before weight predictions under scenario *R*_2_ increase to the point of becoming “too incongruent” with incoming sensory data to be probable, *R*_2_ continues to exert influence on the magnitude of the experienced SWI. According to the model, the *degree* to which an observer believes the smaller object will be denser should exhibit a direct relationship with the individual’s perceived SWI illusion ($\hat {w}$). This prediction was tested and confirmed in Experiment 2.

### Experiment 2: relationship between prior expectations and illusion magnitude

To test this novel prediction, we relied on examination of individual differences. A new group of subjects reported *Expectations* (Expected Weight, EW) about objects’ weight relationships with the instructions that the smaller cube would be denser than the larger by some unspecified amount. These participants then returned within one to two days to undertake *Lifting* sessions and report their the SWI experience (Perceived Weight, PW).

For EW, most subjects reported believing the smaller cube should weigh more than the larger one, for all six box pairs (# of subjects expecting the smaller cube to be heavier: *n*_*A*:*B*_ = 25, *n*_*A*:*C*_ = 23, *n*_*A*:*D*_ = 20, *n*_*B*:*C*_ = 26, *n*_*B*:*D*_ = 22, *n*_*C*:*D*_ = 25). This corresponds to a belief that under *R*_2_, the smaller box is not only denser than the larger one, but by an amount that is so extreme as to make it actually weigh more than the larger one.

For the PW data, a 3 (weight class) × 6 (pair) repeated measures ANOVA on the mean natural log responses revealed the expected main effect of box pair (*F*(5, 145) = 110.282, *p* < .001) and an additional main effect of weight class (*F*(2, 58) = 16.974, *p* < .001). Further, an interaction was revealed between weight and pair (*F*(10, 290) = 8.777, *p* < .001), showing the amount by which size difference (i.e., pair) influences SWI magnitude increases as weight increases as well (Table S3). These results mirror those of Experiment 1.

To evaluate our prediction, we correlated EW reports (Day 1) with PW reports from the same individual (Day 2). This analysis revealed significant positive correlations between expectations under unequal density and perceived SWI magnitude for five of the six box pairs, with the remaining correlation borderline significant (Fig. 2B). On average, those who believed the smaller object was only slightly denser than the larger experienced smaller SWI magnitude, whereas those who believed the smaller object to be much denser than the larger experienced larger SWI magnitude. These results confirm the predictions of our model.

## Discussion

Previous attempts to account for the SWI with Bayesian decision theory had only considered sensory evidence and prior expectations about heaviness—for example positing that an additional “smaller is heavier” prior is created due to the illusion’s occurrence on a previous lift (Buckingham & Goodale, 2010), which cannot account for the illusion on the first lift. Here, we show that percepts of heaviness in the SWI rest not only just on sensory evidence and expectations about heaviness, but also on expectations about density. Bayesian causal inference about the most probable hidden state of the world (the objects’ categorical density relationship) can provide a compelling account for the illusion: the model can account well for data obtained from two experiments in the present study and more in the literature, namely that the magnitude of the illusion grows as the size discrepancy between objects grows (Anderson, 1970; Cross & Rotkin, 1975; Flanagan & Beltzner, 2000; Flanagan et al., 2001; Grandy & Westwood, 2006; Ross & Di Lollo, 1970; Stevens & Rubin, 1970), and that the illusion attenuates and ultimately reverses when observers are trained with small-heavy and large-light objects (Flanagan, Bittner & Johansson, 2008). Importantly, the model also makes a novel prediction that was confirmed by new data (Experiment 2), that density asymmetry expectations positively correlate with experienced SWI magnitude. Taken together, these findings provide compelling support for the proposed Bayesian competitive-prior model accounting for the SWI.

In the proposed model, the competing density hypotheses are qualitative in nature. One may ask why a continuous space should be partitioned into a categorical representation. First, categorical representation of continuous variables has been successful in accounting for human perception. For example, in order to determine the shape of an object, one must first determine its surface reflectance (Yuille & Bülthoff, 1996). But an observer does not care about the exact value of the reflectance, only whether the object is shiny or matte. Likewise, in determining the slant of a surface, the nervous system must determine how much to use compression cues (a circle appearing as an ellipse) (Knill, 2003)—not by deciding *how* “ellipse-like” an object is, but by partitioning the continuous aspect ratio space into ‘circle’ and ‘ellipse’ categories (Knill, 2003). We propose that the brain may represent density in a similarly efficient way. Maintaining a probability distribution over the quantitative density relationship between two objects could require considerable resources, and so instead a simpler categorical representation may be employed: either objects have equal density, or their density is directly or inversely proportional to their volume. That relative density, not absolute density, is important to the SWI is also supported by the recent finding that SWI magnitude does not change whether objects appear to be made of polystyrene or metal (Buckingham & Goodale, 2013).

It should be noted that the present model is not only valid when two objects are being compared side by side, but is also extensible to situations where one is judging the heaviness of just one or more than two objects: even if only a single object is presented, when we lift it we compare its felt heaviness to an implicit or remembered standard for objects of a given size. It has been shown that humans do represent and use such a remembered standard or prototype in estimates of objects’ weights (Peters, Balzer & Shams, 2015). In the present study two objects were used so as to control the size and weight of the standard, as such a remembered standard is also somewhat variable across individuals. Finally, as the magnitudes of illusion reported here are also consistent with others’ findings (e.g., Flanagan, Bittner & Johansson, 2008; Buckingham et al., 2016), the presented model is likely extensible to other sizes, shapes, and numbers of objects.

We also believe a similar competitive prior mechanism may also explain the Material-Weight Illusion (MWI), in which the denser-*looking* of two identically-sized and identical-mass objects is felt to be lighter—for example, a lead-looking cube feels lighter than a polystyrene-looking cube of the same mass. Similarly to the SWI version, the key is the degree of overlap, or “agreement”, between the incoming haptic sensory information and expectations under different density relationship scenarios. Because the sensory information (that they weigh the same) categorically does not agree with the most likely prior (i.e., that polystyrene ought to be significantly less dense than lead and by a very specific amount based on experience, or the equivalent of a very narrow *p*(*w*|*R*_3_)), one of the other possible density relationships must end up being the most probable a posteriori. Further, because the objects possess the same size, their expected weight relationship given equal density *p*(*w*|*R*_1_) will also be extremely certain: they ought to weigh *exactly* the same. In contrast, the expected relationship given that perhaps the polystyrene-looking cube is *denser* than the lead-looking cube (i.e., under *R*_2_) will likely be very uncertain, leading to large overlap or “agreement” between a relatively broad *p*(*w*|*R*_2_) and the incoming haptic sensory information—larger, potentially, than the “agreement” between a noisy haptic likelihood and a *very* certain (narrow) weight expectation given equal density, *p*(*w*|*R*_1_). Such a scenario would lead to the polystyrene-looking cube feeling heavier, or the MWI. Future studies should quantitatively investigate whether the competitive prior framework (outlined here) can indeed account for the MWI, and to explore how competitive priors may explain other weight illusions as well (e.g., effects with familiar objects). Indeed, this computational strategy for representing variables may be a common strategy used by the brain in optimizing efficiency in representation and computation.

Importantly, the proposed model is computationally equivalent to Bayesian causal inference models that have been shown to account for a number of other multisensory perceptual phenomena—including the sound-induced flash illusion (Wozny, Beierholm & Shams, 2008), ventriloquist illusion (Wozny, Beierholm & Shams, 2010), and rubber hand illusion (Samad, Chung & Shams, 2015)—spanning numerous modality combinations and tasks. It is also mathematically similar to other Bayesian competitive prior models that can account for visual perception in very different tasks (Hedges, Stocker & Simoncelli, 2011; Knill, 2003; Knill, 2007; Wozny, Beierholm & Shams, 2008; Yuille & Bülthoff, 1996). Therefore, it appears that SWI is governed by the same computational strategy that has been shown to govern many other perceptual phenomena, meaning this illusion is no longer as esoteric and counter-intuitive as it may have appeared in the past but results from optimal statistical inference. Our findings strongly suggest that even in the realm of counterintuitive and illusory percepts, Bayesian hierarchical causal inference can provide a parsimonious and unifying account of the human perceptual system.

##  Supplemental Information

10.7717/peerj.2124/supp-1 Supplemental Information 1 Supplemental information

10.7717/peerj.2124/supp-2 Data S1 Raw data from Experiments 1 and 2

## Additional Information and Declarations

### Data Availability

The following information was supplied regarding data availability: The raw data has been supplied as Data S1.
