# Supplementary material for: The Size-Weight Illusion is not anti-Bayesian after all: a unifying Bayesian account
Source: PeerJ. 2016 Jun 16;4:e2124. doi: 10.7717/peerj.2124 (PMC4918219; doi:10.7717/peerj.2124)
Supplement: Supplemental Information 1 [file peerj-04-2124-s001.docx]

The Size-Weight Illusion is not anti-Bayesian after all:

A unifying Bayesian account

Megan A. K. Peters, Wei Ji Ma, and Ladan Shams

**Supplemental Information**

**How basic Bayesian accounts fail**

**
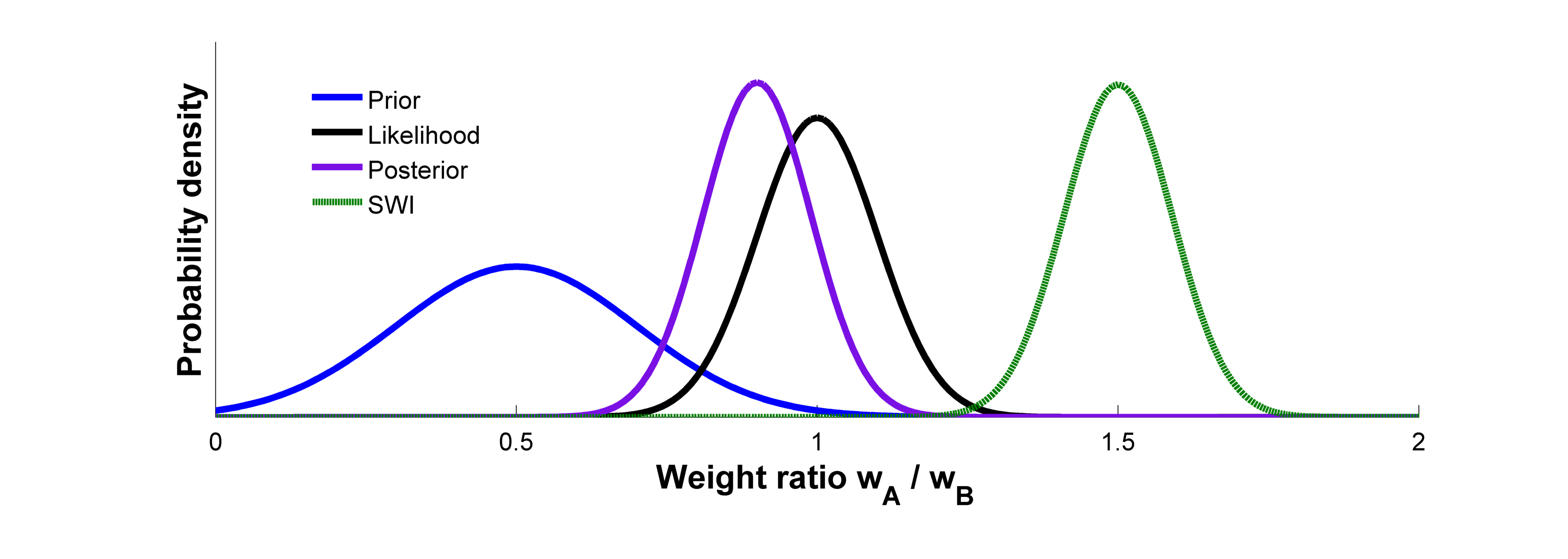
**Consider an example: You are presented with two objects, A and B, that appear to be made of the same material. Because A is smaller than B, you expect it will weigh less than B, i.e. a weight ratio $\frac{w_{A,\mathrm{Expected}}}{w_{B,\mathrm{Expected}}}<1$. However, a clever experimenter has manipulated them such that they possess identical weight, so $\frac{w_{A,\mathrm{Measured}}}{w_{B,\mathrm{Measured}}}=1$. Assuming that we can represent expectations and sensory evidence as a Gaussian prior and a Gaussian likelihood, respectively, we can use Bayes’ rule to obtain a posterior distribution over the weight ratio (Fig. S1). This posterior will peak at a positive value, so A should feel lighter than B. Yet it doesn’t—instead, $\frac{w_{A,\mathrm{Perceived}}}{w_{B,\mathrm{Perceived}}}>1$, so it appears that the expected weight relationship between the two objects (the *prior*) is in the “wrong direction” to produce the SWI.

**Figure S1**. Graphical depiction of the SWI as attempted to be captured by Bayesian inference. With the expectation that the smaller object A will weigh substantially less than the larger object B (*prior*), or $\frac{w_{A,\mathrm{Predicted}}}{w_{B,\mathrm{Predicted}}}<<1$, and the sensory evidence that they weigh the same (*likelihood*), or $\frac{w_{A,\mathrm{Sensory}}}{w_{B, \mathrm{Sensory}}}=1$, Bayes’ rule predicts that A should feel slightly less heavy than B (*posterior*), i.e. that $\frac{w_{A,\mathrm{Perceived}}}{w_{B,\mathrm{Perceived}}}<1$. However, in reality, A will feel heavier than B (*SWI*), i.e. $\frac{w_{A,\mathrm{Perceived}}}{w_{B,\mathrm{Perceived}}}>1$.

**Alternative Models**

For completeness, we test two alternatives to the normative ideal observer model proposed in the main text, which we call $M_{0}$. Notably, neither of the alternative models succeeded in producing the SWI: Both produced predictions consistent with the initial, “anti-Bayesian” formulation of the model proposed prior to this series of studies.

*Alternative Model 1: Changing Axes of Inference*

Although many models have been put forth in which a continuous secondary variable (e.g., surface reflectance) is partitioned into categories (Lambertian vs. specular) in order to utilize the competitive prior framework, the natural question arises as to whether such categorization and hierarchy is truly necessary. Couldn’t inference be done over the continuous space of the secondary variable, and then applied to the primary variable?

In its simplest version, this formulation, which we call $M_{1}$, is essentially a change in axes of the original attempts to explain the SWI with Bayesian inference – those which led to it having been labeled as an “anti-Bayesian” phenomenon. To see how, we define the continuous density space as it was measured in the environment by Peters, Balzer, & Shams (2015) using techniques described in Balzer, Peters, & Soatto (2013), which demonstrates that density follows a power function of volume for liftable, man-made objects. Thus, for the two objects A and B with $V_{A}<V_{B}$, $d_{A}>d_{B}$ on average. However, note that this does *not* indicate in the everyday environment that A weighs more than B. Qualitatively, this presents the same problem as the original Bayesian formulation of the SWI: Even if the incoming sensory information indicates that A is denser than B ($d_{A}>d_{B}$ but $w_{A}=w_{B}$), and this sensory information is combined with priors about the typical density of objects according to Bayes’ rule ($d_{A}>d_{B}$ but $w_{A}<w_{B}$), the posterior suggests that A may be denser than B *but not in the correct direction to make it heavier than B.* Once again, the illusion appears to be “anti-Bayesian” in this formulation.

*Alternative Model 2: Using a Single Continuous Distribution of Density*

While simply using density as a continuous hidden variable and then applying the result to the weight domain does not produce the SWI, it is possible that an alternative formulation of density as a single, continuous secondary variable may succeed. To assess the viability of one such model, we again rely on the environmental data collected by Peters et al. (2015) to form the prior distribution. Kernel density estimates were used to approximate empirical distributions rather than relying on parametric fits which might constrain the probability density functions’ shapes improperly.

This model, $M_{2}$, assesses the weight estimate for each object individually, rather than a pair of objects together. Thus, for each of the two objects A and B, with volumes $V_{A}<V_{B}$ as before, weight $w$ is assessed via Bayes’ rule:

(S11)

with $x_{H}$ defined as the haptic estimate of weight for the single object, and $x_{V}$ defined as the visual estimate of volume for the single object. For $p(w|x_{V}$) we obtain:

(S12)

Because an estimate of weight must be nonnegative, we select a truncated normal distribution to serve as the weight estimate given density and the visual volume estimate with zero probability assigned to negative values for weight, giving $p\left( w | d,x_{V} \right)\sim N\left( \mu,\sigma\right)$, with $\mu=dx_{V}$ and $\sigma=\sigma_{d}$. Then,

(S13)

with $p(d)$ taken as the kernel density estimate of object density frequencies as observed in empirical data (Peters et al., 2015). Finally, for $p(x_{V}|d)$, we obtain:

(S14)

Because a visual estimate of volume must be nonnegative and generally underestimates true volume (i.e., estimated volume is approximately equal to true volume raised to the .704 power) (Frayman & Dawson, 1981), we select a truncated normal distribution for the visual volume estimate with zero probability assigned to negative volumes, such that $p(x_{V}|V)\sim N(V^{.704},\sigma_{V})$.

We tested this model using a Monte Carlo simulation with 1,000 draws from each visual estimate distribution. For a large range of reasonable values for $\sigma_{d}$ and $\sigma_{V}$, the model produced weight estimates for the two objects consistent only with the original, “anti-Bayesian” formulation of the SWI: Smaller objects were predicted to feel lighter than larger objects, consistent with Bayes’ rule but opposing the subjective percept of the SWI itself. Thus, despite relying on density as a hidden factor and marginalizing over it to arrive at the estimates of the variable of interest (i.e., weight), a Bayesian framework which does not include competing categorical hypotheses fails to account for the SWI.

*Another Bayesian Approach*

It was proposed relatively recently that efficient coding can produce biases *away* from the prior distribution in the mean estimate of a posterior distribution, when the likelihood function is skewed (Wei & Stocker, 2012). Although at first it may seem this type of model can produce the anti-Bayesian biases of the SWI, it soon becomes clear that such a model is quite similar to Alternative Model 2, above. With the addition of a skewed likelihood rather than a truncated normal distribution, as was used in the simulation above, although the mean of the posterior distribution is indeed skewed away from the prior in comparison to behavior due to a non-skewed distribution, the qualitative result is the same: the larger object still “feels” heavier than the smaller, which is the opposite of the experienced SWI.

**Supplemental Tables**

**Table S1**. Summary of generative model of SWI.

| **** | **Variable** | **Definition** |
| --- | --- | --- |
|  | $R$ | density relationship between objects A and B |
|  | $v$ | log volume ratio: $v=ln\frac{V_{A}}{V_{B}}$ |
|  | $d$ | log density ratio:$d=ln\frac{d_{A}}{d_{B}}$ |
|  | $w$ | log weight ratio:$w=ln\frac{w_{A}}{w_{B}}$ |
|  | $y$ | log volume measurement ratio:$y=ln\frac{y_{A}}{y_{B}}\sim N(v,\sigma_{y}^{2})$ |
|  | $x$ | log weight measurement ratio:$x=ln\frac{w_{A}}{w_{B}}\sim N(w,\sigma_{x}^{2})$ |

**Table S2**. Means and standard deviations for ln SWI magnitude ($ln(\hat{w}$)) for each pair in Experiment 1.

|  | **Light** | | **Medium** | | **Heavy** | |
| --- | --- | --- | --- | --- | --- | --- |
| **Pair** | $\boldsymbol{\mu}$ | $\boldsymbol{\sigma}$ | $\boldsymbol{\mu}$ | $\boldsymbol{\sigma}$ | $\boldsymbol{\mu}$ | $\boldsymbol{\sigma}$ |
| **A:D** | .3164 | .2491 | .3505 | .2585 | .4168 | .2728 |
| **A:C** | .1392 | .1518 | .1737 | .1557 | .3301 | .3041 |
| **B:D** | .2815 | .2650 | .1602 | .1469 | .2718 | .2681 |
| **A:B** | .0500 | .0697 | .1345 | .1728 | .1995 | .2908 |
| **C:D** | .1668 | .1937 | .1107 | .1421 | .1628 | .2101 |
| **B:C** | .0506 | .0857 | .0869 | .1497 | .1481 | .2332 |

**Table S3**. Means and standard deviations for ln Expected Weight under Unequal Density assumptions (UD) and ln Perceived Weight (PW) in Experiment 2.

| **Condition** | **EW(UD) Mean** | **EW(UD) Std** | **PW Mean** | **PW Std** |
| --- | --- | --- | --- | --- |
| **A:D** | 0.4581 | 0.3174 | 0.3422 | 0.1754 |
| **A:C** | 0.2461 | 0.2162 | 0.2327 | 0.1229 |
| **B:D** | 0.3021 | 0.2684 | 0.2274 | 0.1013 |
| **A:B** | 0.2055 | 0.1489 | 0.1649 | 0.0984 |
| **C:D** | 0.2428 | 0.1852 | 0.1563 | 0.0862 |
| **B:C** | 0.1706 | 0.1215 | 0.1082 | 0.0655 |

**Supplemental References**

Balzer, J., Peters, M. A. K., & Soatto, S. (2013). Volumetric Reconstruction Applied to Perceptual Studies of Size and Weight. In *IEEE Workshop on Applications of Computer Vision (WACV)*.

Frayman, B., & Dawson, W. (1981). The effect of object shape and mode of presentation on judgments of apparent volume. *Perception & Psychophysics*, *29*(1), 56–62.

Peters, M. A. K., Balzer, J., & Shams, L. (2015). Smaller = denser, and the brain knows it: natural statistics of object density drive weight expectations. *PLoS ONE*, *10*, e0119794. doi:10.1371/journal.pone.0119794

Wei, X., & Stocker, a. (2012). Efficient coding provides a direct link between prior and likelihood in perceptual Bayesian inference. *Advances in Neural Information …*, 1–9.
